# Neurocognitive and Psychosocial Interactions in Atrial Fibrillation: Toward a Holistic Model of Care

**DOI:** 10.3390/healthcare13151863

**Published:** 2025-07-30

**Authors:** Tunde Pal, Zoltan Preg, Dragos-Florin Baba, Dalma Balint-Szentendrey, Attila Polgar, Csilla-Gerda Pap, Marta German-Sallo

**Affiliations:** 1 Department of Internal Medicine V, George Emil Palade University of Medicine Pharmacy, Science, and Technology of Targu Mures, Gheorghe Marinescu 38, 540142 Targu Mures, Romania; 2 Cardiovascular Rehabilitation Clinic, County Emergency Clinical Hospital of Targu Mures, Gheorghe Marinescu 50, 540136 Targu Mures, Romania; zoltan.preg@umfst.ro (Z.P.); szentendreydalma@gmail.com (D.B.-S.); marta.german-sallo@umfst.ro (M.G.-S.); 3 Department of Family Medicine, George Emil Palade University of Medicine Pharmacy, Science, and Technology of Targu Mures, Gheorghe Marinescu 38, 540142 Targu Mures, Romania; 4 Department of Cell and Molecular Biology, George Emil Palade University of Medicine Pharmacy, Science, and Technology of Targu Mures, Gheorghe Marinescu 38, 540142 Targu Mures, Romania; dragos-florin.baba@umfst.ro; 5 Emergency Institute for Cardiovascular Diseases and Transplantation of Targu Mures, Gheorghe Marinescu 50, 540136 Targu Mures, Romania; 6 Faculty of Medicine, George Emil Palade University of Medicine Pharmacy, Science, and Technology of Targu Mures, Gheorghe Marinescu 38, 540142 Targu Mures, Romania; polgar.attila.20@stud.umfst.ro (A.P.); pap.csilla-gerda@stud19.umfst.ro (C.-G.P.); 7 Department of Internal Medicine III, George Emil Palade University of Medicine Pharmacy, Science, and Technology of Targu Mures, 540142 Targu Mures, Romania

**Keywords:** atrial fibrillation, mental health, psychosocial factor, cognitive dysfunction: psychosocial distress, cardiovascular disease, quality of life in atrial fibrillation, cardiovascular rehabilitation

## Abstract

**Background/Objectives:** Psychosocial (PS) factors and cognitive dysfunction (CD) in patients with atrial fibrillation (AF) may negatively impact treatment compliance. The PS profile covers multiple psychological and socio-economic factors, although research is mostly limited to depression, anxiety, and work stress. This study assessed the prevalence of a broad range of PS factors in patients with AF and their relationship with cognitive decline. **Methods:** We retrospectively analyzed data from patients referred to a cardiovascular rehabilitation clinic between March 2017 and April 2023 who underwent standardized assessments of PS factors, cognition, and quality of life. **Results:** Of the 798 included patients, 230 (28.8%) had AF, with a mean age of 68.07 years (SD 9.60 years). Six of nine PS factors were present in more than half of the overall sample. Compared to non-AF patients, those with AF showed significantly higher levels of social isolation, depression, and hostility, whereas low socioeconomic status, family and work-related stress, and other mental disorders were more frequent in the non-AF group. CD was present in 67.4% of the total cohort and was more prevalent in AF patients with a higher PS burden. Patients with permanent AF reported the poorest health status. **Conclusions:** Integrating assessments of PS factors and cognition in cardiac rehabilitation is feasible and supports a more comprehensive, patient-centred model of care in AF.

## 1. Introduction

As the population ages, atrial fibrillation (AF) shows a continuous rise in incidence [1,2] and, partly for the same reason, dementia also shows a rise in new cases [3,4]. They represent an emerging concern as both contribute to increased health, economic, and psychosocial burdens on caregivers [2,3]. While the real number of cases is unknown, AF could be asymptomatic [5] and cognitive dysfunction (CD) is diagnosed in several cases too late [6]. Prior studies suggested a close relationship between AF and the incidence of CD [7,8,9,10], though there are still gaps in the evidence regarding the pathomechanism and potential pathways, blood-based and brain imaging biomarkers, and prevention strategies (the role of oral anticoagulation therapy, and the benefits of rhythm versus rate control) [11].

Mood disorders, such as depressive symptoms and stress, negatively affect general health, quality of life, lifestyle choices, acceptance of illness and treatment adherence [12,13,14]. Psychosocial (PS) distress may influence both the incidence and prognosis of AF [15,16]; however, robust evidence remains limited [17,18]. Depression, anxiety, and work stress are probably the most studied research questions [15,17]. Previous research indicates that anxiety and depression frequently co-occur in patients with AF [19]. Furthermore, PS stressors are associated with CD and dementia, with evidence supporting a bidirectional relationship. Some of these stressors, such as depression, were identified as modifiable risk factors for CD [20] and may also contribute to the development or progression of dementia [21].

PS factors are getting into the spotlight as a significant emerging area of research in cardiology, particularly in relation to the incidence, severity, and prognosis of AF. Building on prior findings, the systematic assessment and management of PS stressors should be integrated into the comprehensive care of patients with AF and implemented in cardiac rehabilitation programmes [22,23]. The current conceptualization of PS risk factors, however, often remains narrowly focused on commonly studied domains such as anxiety, depression, or work-related stress [24]. Consequently, the literature addressing a broader spectrum of PS stressors in the context of AF remains limited. This study aims to address this gap by evaluating the prevalence of a broad range of PS factors in a middle-aged and older population with AF and examining their association with cognitive decline in AF.

## 2. Materials and Methods

This study is part of a single-centre, cross-sectional study conducted at a cardiovascular rehabilitation clinic. The primary objective was to evaluate the prevalence of CD among patients with cardiovascular diseases (CVDs). The STROBE cross-sectional checklist applicable to this study can be found in Supplementary Table S1.

### 2.1. Study Population

We retrospectively analyzed data from patients referred to the clinic between March 2017 and April 2023 for various cardiovascular or metabolic complaints, further diagnostic evaluation, treatment optimization, and participation in a cardiovascular rehabilitation programme.

Clinical data, including personal and family history, cardiovascular risk factors, diagnoses of cardiovascular diseases, and comorbid conditions, were obtained through medical record review. Patients were included if they had a confirmed diagnosis—either pre-existing or established during hospitalization—of AF, chronic coronary syndrome, chronic heart failure, or peripheral artery disease. AF was identified based on documented history or the presence of arrhythmia on a standard 12-lead electrocardiogram. Exclusion criteria included refusal to participate or lack of informed consent, acute disease phases, recent major cardio or cerebrovascular events, severe renal impairment (on dialysis, estimated glomerular filtration rate < 15 mL/min/1.73 m^2^) and diagnosed Alzheimer’s disease, or other types of dementia. Patients with blindness, deafness, or motor deficits (e.g., paresis or paralysis of the dominant hand) that could interfere with cognitive assessment were also excluded.

### 2.2. Cognitive Evaluation

In this study, we aimed to investigate any degree of change in cognitive decline, and we chose the Montreal Cognitive Assessment (MoCA) test, the standard paper form. This cognitive battery has great specificity and sensitivity to detect mild CD compared to other tests, such as the Mini-Mental State Examination, which was developed for the detection of dementia [25,26,27,28,29]. It is a validated and widely used brief cognitive screening tool in cardiology and neurology research [26,27,30,31,32] that enables us to make valid and relevant comparisons of the study findings. The MoCA evaluates multiple cognitive domains, including the following: visuospatial/executive (5 points), naming (3 points), attention (6 points), language (3 points), abstraction (2 points), delayed recall (5 points), and orientation (6 points). The maximum possible score is 30 points, with a cut-off score of <26 indicating CD [33]. In accordance with standard MoCA scoring guidelines, one additional point was awarded to participants with fewer than 12 years of formal education. For the purposes of this study, the conventional cut-off score of 26 was used to define CD.

### 2.3. Evaluation of Psychosocial Risk Factors

The 2016 European Guideline on Cardiovascular Disease Prevention recommends the assessment of PS risk factors in selected populations, recognizing their impact on the burden of CVDs, as well as on lifestyle-related behaviours and treatment adherence [34]. We needed a test that explores a wider range of factors, is easy to administer, and does not need special skills or tuition. As far as we know, the questionnaire recommended by the European Society of Cardiology evaluates the most components of psychological and socio-economic well-being, which is used in CVDs. Other instruments evaluate a limited number of factors, such as the Hospital Anxiety and Depression Scale or Beck Anxiety and Depression Inventory [35], or they were developed for special circumstances, such as the Copenhagen Psychosocial Questionnaire applied at the workplace [36]. PS stressors are considered important risk modifiers in cardiovascular risk stratification, alongside other factors such as family history of premature CVDs, central obesity and body mass index, coronary calcium score (assessed via computed tomography), ankle–brachial index, and carotid ultrasound detection of atherosclerotic plaques [34]. To evaluate these risk factors, participants completed the standardized self-administered psychosocial questionnaire developed by the European Society of Cardiology [34]. The questionnaire, which takes less than 10 min to complete, includes 19 items assessing nine psychosocial and socioeconomic domains: low socio-economic status (including one question on educational attainment), work-, and family-related stress, social isolation, depression, anxiety, hostility, type D personality, post-traumatic stress disorder, and other mental health disorders. Education level is categorized into six groups: 1st Category: 1–4 classes, 2nd Category: 5–8 classes, 3rd Category: Gymnasium, 4th Category: Professional school, 5th Category: Vocational school and6th Category: University degree. All PS factors—except for education—were assessed using binary (yes/no) responses, and a single “yes” response was sufficient to classify a patient as positive for that particular PS factor [34].

### 2.4. Evaluation of Quality of Life

In AF, apart from the evaluation of cognitive, physical, and emotional well-being, quality of life assessment is also part of the patient-reported outcome measures. Generally, health-related quality of life questionnaire is encouraged, but some of them involve financial aspects and need licence in order to use it. A well-used instrument is the Short-Form 12 quality of life questionnaire or disease-specific Atrial Fibrillation Effect on Quality of life (AFEQT) [37]. For this study, official permission for using the EQ-5D-5L questionnaire was obtained. It is a validated, self-administered instrument designed to measure health-related quality of life across five dimensions: mobility, self-care, usual activities, pain/discomfort, and anxiety/depression. Each dimension is rated on five levels of severity, ranging from no problems to extreme problems. In addition to the descriptive system, the EQ-5D-5L includes a visual analogue scale where participants rate their overall health status on the day of assessment on a scale from 0 to 100, with 0 representing the worst imaginable health state and 100 the best [38]. Both the EQ-5D-5L and earlier versions of the instrument are widely used in cardiovascular research and clinical practice to evaluate patient-reported outcomes in individuals with CVDs [39,40,41,42].

### 2.5. Statistical Methods

Statistical analysis was conducted using MedCalc Software, version 19. Descriptive statistics were used to summarize baseline characteristics, with results expressed as means ± standard deviations (SD) for continuous variables and percentages for categorical variables. The Shapiro–Wilk test was applied to assess the normality of data distributions [43]. Group comparisons were performed using the Student *t* test for normally distributed (parametric) data, the Mann–Whitney U test for (non-normally distributed) data, and the chi-square (χ^2^) test for categorical variables. Statistical significance was defined as a *p* value less than 0.05.

## 3. Results

A total of 798 patients were included in the final analyses; 89 patients of the whole sample were not considered as they lacked data regarding MoCA test results or cognitive domain results (Table 1). The mean age was 68.07 years ± 9.60 years, and 50.5% of the participants were female. The majority (58.5%) resided in urban areas, and the mean educational attainment was approximately 11 years of formal education. The subgroup with AF included 230 patients (28.8%). Compared to those without AF, patients in the AF group had a lower prevalence of traditional cardiovascular risk factors but exhibited higher rates of heart failure, chronic kidney disease, and prior stroke.

### 3.1. Psychosocial Risk Profile

Psychosocial stressors were highly prevalent across both study groups. Out of the nine assessed PS factors, six were present in more than half of the total population. Among patients with AF, the most frequently reported stressors were social isolation (83.5%; *n* = 147), followed by low socio-economic status (73.9%; *n* = 119), hostility (60.8%; *n* = 107), and work- and family-related stress (60.6%; *n* = 86). Statistically significant differences were observed between groups. In the AF group, social isolation (*p* < 0.0001), depression (*p* < 0.0001), and hostility (*p* = 0.0053) were significantly more common. In contrast, patients without AF more frequently reported low socio-economic status (*p* < 0.0001), work- and family-related stress (*p* = 0.0149), and other mental disorders (*p* < 0.0001) (Table 2). Regarding gender, except for other mental health disorders, we identified no significant difference between gender and PS factors or between AF status and gender (Table 3).

### 3.2. Cognitive Evaluation and Psychosocial Stressors

The overall prevalence of cognitive decline was 67.4% (*n* = 539). Although the proportion of patients with CD did not differ significantly between those with AF and those in sinus rhythm (73.5% vs. 64.8%; *p* = 0.9131), the mean MoCA scores were significantly lower in the AF group (22.52 ± 4.68 vs. 23.24 ± 4.47; *p* = 0.0320). We found a significantly lower score in the AF group in relation to the Visuospatial/Executive domain (*p* = 0.0056). (Table 4).

**Figure 1:**
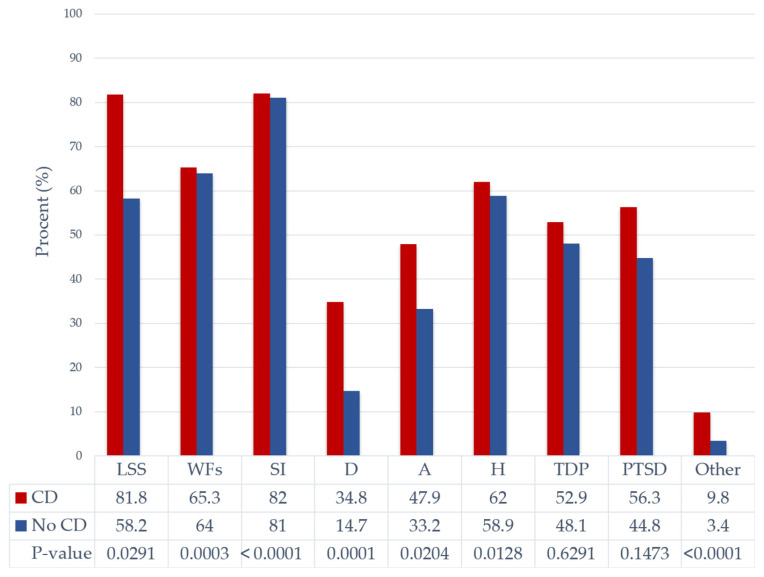
Distribution of psychosocial stressors by cognitive dysfunction. Abbreviations: A, anxiety; CD, cognitive dysfunction; D, depression; H, hostility; LSS, low socioeconomic status; Other, other mental disorders; PTSD, post-traumatic stress disorder; SI, social isolation; TDP, type D personality; and WFs, work and family-related stress.

**Figure 2:**
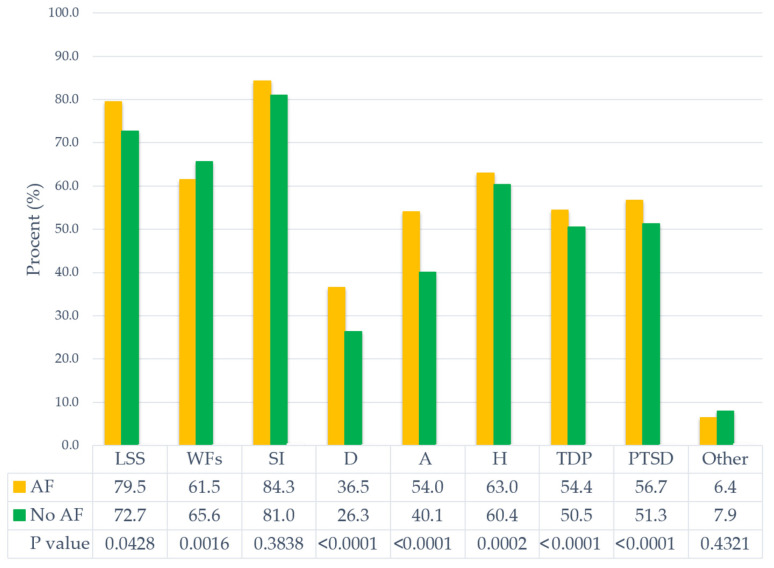
Distribution of psychosocial stressors in patients with cognitive decline according to the presence or absence of atrial fibrillation. Abbreviations: A, anxiety; AF, atrial fibrillation; CD, cognitive dysfunction; D, depression; H, hostility; LSS, low socioeconomic status; Other, other mental disorders; PTSD, post-traumatic stress disorder; SI, social isolation; TDP, type D personality; and WFs, work and family-related stress.

We further analyzed the prevalence of PS stressors among patients with CD regardless of AF status. PS factors were significantly more prevalent with CD, except for type D personality and post-traumatic stress disorders (Figure 1 and Figure 2).

In the subgroup of patients with AF and confirmed CD, several PS factors were significantly more frequent, including the following: low socio-economic status (*p* = 0.0428), depression (*p* < 0.0001), anxiety (*p* < 0.0001), hostility (*p* = 0.0002), type D personality (*p* < 0.0001), and post-traumatic stress disorder (*p* < 0.0001).

### 3.3. Assessment of Quality of Life

Patients with AF reported a lower perceived quality of life on the visual analogue scale of the EQ-5D-5L questionnaire; however, the difference was not statistically significant when compared to patients without AF (Table 5).

Among AF subtypes, individuals with permanent AF reported the poorest health status, as reflected in both the EQ-5D-5L dimension scores and visual analogue scale ratings, compared to those with persistent and paroxysmal AF. Details are presented in Table 6.

## 4. Discussion

This study aimed to investigate the PS risk profile of patients with AF and cognitive decline, recognizing the growing importance of comprehensive and personalized care in CVDs. Our observational findings indicate a high prevalence of PS stressors in patients with CVDs. Notably, patients with AF more frequently reported social isolation, depressive symptoms, and hostility. Conversely, low socio-economic status, work- and family-related stress, and other mental health disorders were more prevalent in patients without AF. Among patients with CD, all PS stressors—except post-traumatic stress disorder and type D personality—were significantly more common. When CD co-occurred with AF, the burden of PS stressors further increased. Specifically, individuals with both AF and CD were more likely to experience low socio-economic status, depression, anxiety, hostility, type D personality, and post-traumatic stress disorder. Quality of life, assessed using the EQ-5D-5L, was lower among patients with AF, particularly in those with permanent AF. Although this finding was not statistically significant between AF and non-AF groups overall, the subgroup analysis of AF types suggested a gradient of impairment.

Contemporary cardiovascular medicine emphasizes a holistic and individualized approach to AF management; however, implementing such comprehensive care in routine practice remains challenging [22]. This includes the integration of psychological and social factors into cardiac rehabilitation and even into rhythm management strategies. The assessment of cognitive abilities is also endorsed during rehabilitation, including for patients with AF [18,44]. Although the potential negative impact of PS stressors on AF incidence and outcomes has been increasingly recognized and current guidelines encourage the assessment of PS factors [18], clinical attention remains disproportionately focused on depression and anxiety, with other stressors often overlooked [15]. Our study attempts to bridge this gap by evaluating nine PS domains in relation to AF and cognitive decline.

PS stressors are known to influence health behaviours, CVD risk, and AF pathogenesis [12,23]. However, prior research has disproportionately focused on coronary disease, often neglecting conditions such as AF [45,46]. In our cohort, the PS profiles of patients with and without AF differed significantly. These findings are consistent with the SAGE-AF study, where nearly 60% of patients with AF had at least one of the following: depression, anxiety, or cognitive impairment. Additionally, the clustering of these conditions was strongly associated with reduced quality of life and higher symptom burden [19]. We also found that depressive symptoms, along with hostility and social isolation, were more frequent in patients with AF. In addition to psychological distress, the presence of multiple PS stressors in AF patients with CD might be linked to underlying systemic inflammation, autonomic dysfunction, or dysregulation of the renin–angiotensin–aldosterone and hypothalamic–pituitary–adrenal axes [24,47,48]. These biological pathways have been implicated in both AF and neurocognitive decline [49]. Previous studies demonstrated a close relationship between stress and the incidence of AF, as a higher degree of stress was associated with AF incidence [15,50]. Interestingly, our results diverged from some previous studies. For instance, work- and family-related stress and low socioeconomic status were less common among AF patients in our sample, contrary to reports associating these stressors with a higher AF burden [16]. The same pattern was observed when AF and cognitive decline co-occurred, as these specific stressors remained less prevalent compared to patients without known AF. These unexpected findings may reflect selection biases, unmeasured confounders, differential perception/reporting of stress across subgroups, or health system context.

Although our study did not specifically investigate hospitalization outcomes, previous research has highlighted the clinical consequences of psychosocial stressors in AF. For example, Meyre et al. demonstrated that patients experiencing social isolation were at a significantly higher risk of unplanned hospitalizations, whereas depression and low educational level were not independently associated. These findings underline the broader impact of PS factors on healthcare utilization and further support the need for their assessment in comprehensive AF management [51].

Regarding CD, patients with AF status had demonstrated a higher prevalence of PS stressors compared to patients without impaired cognitive abilities. Previous research has demonstrated that PS factors such as depression, post-traumatic stress disorder, anxiety, stress, anger, socioeconomic status, poor social support, and type D personality are all associated with AF [52]. Our findings are consistent with these associations, as we also identified a high prevalence of depressive symptoms, social isolation, and hostility in AF patients. Notably, the number of PS stressors further increased in patients who also had cognitive decline, indicating a cumulative burden in this subgroup. On the other hand, patients with AF in our study were older and had a higher prevalence of heart failure, chronic kidney disease, and stroke conditions that have independently been linked to PS factors in earlier studies [46]. These comorbidities may contribute to or amplify the psychosocial vulnerability observed in this population. In patients with both AF and CD, the clustering of stressors such as depression, hostility, and anxiety may be multifactorial. For example, socioeconomic deprivation and limited educational attainment are well-established risk factors for cognitive impairment and dementia [3,18]. Therefore, while CD may exacerbate vulnerability to PS stress, some stressors may also act as antecedents of cognitive deterioration.

Additionally, our study found that social isolation was remarkably prevalent across all subgroups. This aligns with broader public health concerns, as social isolation has been recognized by the World Health Organization as a major risk factor for cognitive decline, dementia, and even mortality [53,54,55]. The older age of AF patients in our sample likely contributed to their increased levels of social isolation. The mean age in this sample was 68 years, which is considered old; however, it is not so high compared to other studies on cognitive decline.

Contrary to several earlier reports suggesting that women are more vulnerable to certain PS stressors, such as depression or post-traumatic stress disorder [56], our study found no significant gender differences. These findings align with more recent investigations that challenge traditional assumptions about sex-based disparities in psychological stress burden [48].

Cardiovascular diseases reduce the quality of life [57], and its evaluation is feasible, even in routine clinical and rehabilitation settings [23]. Despite the fact that quality of life was assessed in a smaller proportion of patients in our study, and we used a generic rather than disease-specific tool, such as the AFEQT questionnaire, our findings suggest that patients with AF experience a lower perceived quality of life compared to those without AF. This was especially observed in individuals with permanent AF, likely due to their higher comorbidity index. These findings emphasize the importance of including quality of life assessments in the holistic care of AF patients, particularly within cardiac rehabilitation programmes.

This study makes a meaningful contribution to the limited literature on the broader psychosocial landscape in AF. Most prior studies have focused narrowly on depression and anxiety, whereas we evaluated a wider range of stressors and linked them to both AF and cognitive function. Our findings underscore the need for clinicians to consider a more comprehensive PS assessment in AF management, particularly when CD is present. Nevertheless, some of the differences observed—such as the 0.7-point reduction in MoCA scores—achieved statistical significance, the clinical relevance of these findings must be interpreted with caution. Such differences, though modest, may still reflect early cognitive decline or contribute to a cumulative burden when considered alongside other psychosocial and health-related factors. However, we acknowledge that these effect sizes may not independently indicate clinically meaningful impairment, and future studies should aim to establish clinically relevant thresholds to guide interpretation.

Finally, several potential limitations must be taken into consideration. First, the assessment of cognitive decline, PS distress, and quality of life was limited to single, validated instruments. We supported questionnaire choices in an earlier section of this manuscript. While widely accepted, these tools might underestimate or overestimate the true prevalence of these conditions. However, the MoCA questionnaire is a well-validated cognitive screening battery, and the applied psychosocial questionnaire is recommended by the European Society of Cardiology [29,34,58]. Second, both the PS profile and CD were evaluated at a single time point, limiting our ability to determine causality or temporal sequence. A follow-up evaluation in out-of-hospital settings would strengthen these findings. Third, the cross-sectional nature of this study also prevents establishing whether PS stressors predispose to AF or CD, or if these conditions themselves intensify psychosocial burden. Fourth, unmeasured variables such as medication adherence, severity of comorbid conditions, or social support networks, but also age, could have influenced the observed associations. Analysis of these confounding factors in a multivariate analysis was not performed; however, it would have been warranted. Fifth, patients admitted to a rehabilitation clinic might have multiple comorbidities and altered functional capacity, which can influence emotional and physical well-being. There would have been a rational comparison of our findings with a control group from the general population or other non-cardiac patients. Finally, although our sample size was moderate, further replication in larger and more diverse populations is warranted to strengthen external validity.

## 5. Conclusions

The findings of this study suggest that PS stressors are highly prevalent among patients with AF, extending beyond the commonly examined domains of depression, anxiety, and stress. Factors such as social isolation, hostility, and socioeconomic status also appear to be relevant and may contribute to the overall burden experienced by this patient population. Furthermore, the co-occurrence of CD with AF may intensify these stressors, reflecting a complex interaction between psychological, cognitive, and cardiovascular factors.

While our cross-sectional data do not permit causal conclusions, these associations highlight the potential value of broadening the scope of psychosocial and cognitive assessments in AF populations. Future longitudinal studies are warranted to confirm these findings and evaluate the clinical impact of incorporating such assessments into routine care. In the context of cardiac rehabilitation, a more holistic and individualized approach may ultimately enhance patient-centred management strategies for AF.

## Figures and Tables

**Table 1:** Demographic and clinical characteristics of participants.

	Total (*n* = 798) 100%	AF (*n* = 230) 28.8%	No AF (*n* = 568) 71.2%
Age, yrs (mean, SD)	68.07 (9.60)	72.33 (8.06)	66.35 (9.65)
Females (n, %)	403 (50.5)	116 (50.4)	287 (50.5)
Urban (n, %)	467 (58.5)	131 (57.0)	336 (59.2)
Education level, classes (mean, SD)	10.91 (2.88)	10.72 (3.07)	10.99 (2.80)
BMI, kg/m^2^ (mean, SD)	30.66 (6.02)	30.06 (5.11)	30.90 (6.35)
MoCA, pts (mean, SD)	23.03 (4.54)	22.52 (4.68)	23.24 (4.47)
CD (MoCA < 26 points) (n, %)	537 (67.3)	169 (73.5)	368 (64.8)
SBP, mmHg (mean, SD)	133.92 (18.88)	129.97 (18.13)	135.56 (18.96)
DBP, mmHg (mean, SD)	80.68 (10.71)	78.73 (10.21)	81.50 (10.82)
Heart rate, bpm (mean, SD)	73.17 (15.36)	79.01 (20.01)	70.70 (12.09)
Arterial hypertension (n, %)	747 (93.6)	209 (90.9)	538 (94.7)
Type 2 diabetes (n, %)	354 (44.4)	98 (42.6)	256 (45.1)
Dyslipidemia (n, %)	474 (59.4)	112 (48.7)	362 (63.7)
HF (n, %)	429 (53.8)	158 (68.7)	271 (47.7)
IHD (n, %)	297 (37.2)	72 (31.3)	225 (39.6)
CKD (n, %)	446 (55.9)	146 (63.5)	300 (52.8)
Stroke (n, %)	91 (11.4)	30 (13.0)	61 (10.7)

Abbreviations: AF, atrial fibrillation; bpm, beats per minute; BMI, body mass index; CD, cognitive dysfunction; CKD, chronic kidney disease; DBP, diastolic blood pressure; HF, heart failure; IHD, ischemic heart disease; MoCA, Montreal Cognitive Assessment; pts, points; SBP, systolic blood pressure; SD, standard deviation.

**Table 2:** Comparison of psychosocial stressors between AF and non-AF patients.

Psychosocial Risk Factor	AF	No AF	*p* Value
LSS	*119 (73.9)*	*311 (74.2)*	<*0.0001*
WFs	*86 (60.6)*	*257 (66.2)*	*0.0149*
SI	*147 (83.5)*	*361 (80.8)*	<*0.0001*
D	*56 (31.8)*	*118 (26.4)*	<*0.0001*
A	85 (48.6)	179 (40.6)	0.7624
H	*107 (60.8)*	*271 (58.3)*	*0.0053*
TDP	89 (51.1)	228 (51.2)	0.8201
PTSD	95 (54.0)	231 (51.7)	0.3271
Other	*9 (5.2)*	*38 (8.6)*	<*0.0001*

We utilize the chi-square test. Abbreviations: AF, atrial fibrillation; A, anxiety; D, depression; H, hostility; LSS, low socioeconomic status; Other, other mental disorders; PTSD, post-traumatic stress disorder; SI, social isolation; TDP, type D personality; WFs, work and family-related stress.

**Table 3:** Gender-based distribution of psychosocial stressors in AF and non-AF groups.

	Total Sample			Female			Male		
Psychosocial Risk Factor	Female	Male	*p* Value	AF	No AF	*p* Value	AF	No AF	*p* Value
LSS	214 (68.2)	216 (68.4)	0.2127	59 (64.1)	155 (69.8)	0.7887	60 (68.2)	156 (68.4)	0.2363
WFs	169 (53.8)	174 (55.1)	0.7149	45 (48.9)	124 (55.9)	0.9781	41 (46.6)	133 (58.3)	0.4786
SI	268 (85.4)	240 (75.9)	0.7852	78 (84.8)	190 (85.6)	0.2577	69 (78.4)	171 (75.0)	0.7261
D	127 (40.4)	47 (14.9)	0.1466	41 (44.6)	86 (38.7)	0.6919	15 (17.0)	32 (14.0)	0.5772
A	172 (54.8)	92 (29.1)	0.8442	53 (57.6)	119 (53.6)	0.9177	32 (36.4)	60 (26.3)	0.7492
H	204 (65.0)	174 (55.1)	0.5379	57 (62.0)	147 (66.2)	0.8102	50 (56.8)	124 (54.4)	0.7041
TDP	185 (58.9)	132 (41.8)	0.6020	52 (56.5)	133 (59.9)	0.8092	37 (42.0)	95 (41.7)	0.9398
PTSD	199 (63.4)	127 (40.2)	0.9770	61 (66.3)	138 (62.2)	0.6938	34 (38.6)	93 (40.8)	0.6867
Other	*33* *(10.5)*	*14* *(4.4)*	*0.0005*	7 (7.6)	26 (11.7)	0.7718	2 (2.3)	12 (5.3)	0.5026

We utilize the chi-square test. Abbreviations: AF, atrial fibrillation; A, anxiety; D, depression; H, hostility; LSS, low socioeconomic status; Other, other mental disorders; PTSD, post-traumatic stress disorder; SI, social isolation; TDP, type D personality; and WFs, work and family-related stress.

**Table 4:** Scores in patients with and without atrial fibrillation.

Cognitive Domains	AF	No AF	*p* Value
Visuospacial/Executive	*3.26 (1.39)*	*3.56 (1.32)*	*0.0056* **
Naming	2.85 (0.44)	2.83 (0.47)	0.4738 **
Attention	4.61 (1.53)	4.62 (1.47)	0.9536 **
Language	1.84 (0.95)	1.95 (0.98)	0.1048 **
Abstraction	1.41 (0.77)	1.54 (0.68)	0.0572 **
Delayed recall	2.23 (1.65)	2.27 (1.65)	0.7530 **
Orientation	5.82 (0.47)	5.79 (0.63)	0.9634 **

** Mann–Whitney U test. Abbreviations: AF, atrial fibrillation.

**Table 5:** Quality of life in patients with and without atrial fibrillation.

	AF	No AF	*p* Value
EQ-5D-5L Score	22,707.29 (11,749.44)	23,144.75 (11,163.97)	0.9089 **
EQ-5D-5L Scale	65.43 (18.57)	71.95 (18.30)	0.0765 **

** Mann–Whitney U test. Abbreviations: AF, atrial fibrillation; EQ-5D-5L, EuroQol 5-level, 5-dimensional questionnaire used to evaluate quality of life.

**Table 6:** Quality of life across atrial fibrillation subtypes.

	Paroxysmal	Persistent	Permanent	*p* Value
EQ-5D-5L Score	*18,597.19 (9770.67)*	21,111.00 (-)	*28,201.56 (12,538.50)*	*0.0176* *
EQ-5D-5L Scale	*71.75 (16.16)*	- (-)	*57.00 (18.69)*	*0.0061* **

* Student *t* test. ** Mann–Whitney U test. Abbreviations: EQ-5D-5L, EuroQol 5-level, 5-dimensional questionnaire used to evaluate quality of life.

## Data Availability

The study data will be available upon reasonable request to the corresponding author.

## Supplementary Materials

The following supporting information can be downloaded at https://www.mdpi.com/article/10.3390/healthcare13151863/s1, Table S1: STROBE Checklist.

## Abbreviations

The following abbreviations are used in this manuscript:

AF	Atrial fibrillation
CD	Cognitive dysfunction
CVDs	Cardiovascular diseases
MoCA	Montreal Cognitive Assessment
