# Supplementary material for: Neurocognitive and Psychosocial Interactions in Atrial Fibrillation: Toward a Holistic Model of Care
Source: Healthcare (Basel). 2025 Jul 30;13(15):1863. doi: 10.3390/healthcare13151863 (PMC12346523; doi:10.3390/healthcare13151863)
Supplement: Supplementary file 1 [file healthcare-13-01863-s001.zip › healthcare-3701271-supplementary.pdf]

Supplementary Table S1

STROBE Checklist

	Recommendation	Our manuscript
**Title and abstract**	(*a*) Indicate the study's design with a commonly used term in the title or the abstract	Not applicable
	(*b*) Provide in the abstract an informative and balanced summary of what was done and what was found	See abstract, Page 1.
**Introduction**		
Background/rationale	Explain the scientific background and rationale for the investigation being reported	Page 1-2, Introduction section - Paragraph 1, 2 explains scientific background, Paragraph 3 explains rationale
Objectives	State specific objectives, including any prespecified hypotheses	Paragraph 3, Page 2
**Methods**		
Study design	Present key elements of study design early in the paper	Page 2, Material and Methods section, 1st paragraph
Setting	Describe the setting, locations, and relevant dates, including periods of recruitment, exposure, follow-up, and data collection	Page 2, Methods section, 2nd paragraph
Participants	(*a*) Give the eligibility criteria, and the sources and methods of selection of participants	Page 2, Material and Methods, 3rd paragraph
Variables	Clearly define all outcomes, exposures, predictors, potential confounders, and effect modifiers. Give diagnostic criteria, if applicable	Page 2-4 Paragraph 4-6
Data sources/ measurement	For each variable of interest, give sources of data and details of methods of assessment (measurement). Describe comparability of assessment methods if there is more than one group	Page 2-4 Paragraph 4-6
Bias	Describe any efforts to address potential sources of bias	Not applicable
Study size	Explain how the study size was arrived at	Page 4, 1st paragraph of Results section

Quantitative variables	Explain how quantitative variables were handled in the analyses. If applicable, describe which groupings were chosen and why	See Statistical Methods section
Statistical methods	(*a*) Describe all statistical methods, including those used to control for confounding	See Statistical Methods section
	(*b*) Describe any methods used to examine subgroups and interactions	Not applicable
	(*c*) Explain how missing data were addressed	Not applicable
	(*d*) If applicable, describe analytical methods taking account of sampling strategy	Not applicable
	(*e*) Describe any sensitivity analyses	Not applicable
**Results**		
Participants	(a) Report numbers of individuals at each stage of study—eg numbers potentially eligible, examined for eligibility, confirmed eligible, included in the study, completing follow-up, and analysed	Not applicable
	(b) Give reasons for non-participation at each stage	Not applicable
	(c) Consider use of a flow diagram	Not applicable
Descriptive data	(a) Give characteristics of study participants (eg demographic, clinical, social) and information on exposures and potential confounders	Page 4, Results section, 1st paragraph and Table 1.
	(b) Indicate number of participants with missing data for each variable of interest	Not applicable
Outcome data	Report numbers of outcome events or summary measures	Not applicable
Main results	(*a*) Give unadjusted estimates and, if applicable, confounder-adjusted estimates and their precision (eg, 95% confidence interval). Make clear which confounders were adjusted for and why they were included	Page 5-8
	(*b*) Report category boundaries when continuous variables were categorized	Not applicable

	(*c*) If relevant, consider translating estimates of relative risk into absolute risk for a meaningful time period	Not applicable
Other analyses	Report other analyses done—eg analyses of subgroups and interactions, and sensitivity analyses	Not applicable
**Discussion**		
Key results	Summarise key results with reference to study objectives	Page 8, Discussion section, 1st paragraph
Limitations	Discuss limitations of the study, taking into account sources of potential bias or imprecision. Discuss both direction and magnitude of any potential bias	Page 10, Discussion section, paragraph 10
Interpretation	Give a cautious overall interpretation of results considering objectives, limitations, multiplicity of analyses, results from similar studies, and other relevant evidence	Page 10, Conclusion section
Generalisability	Discuss the generalisability (external validity) of the study results	Page 10, Discussion section, paragraph 9
Other information		
Funding	Give the source of funding and the role of the funders for the present study and, if applicable, for the original study on which the present article is based	Funding section, Page 11
